# Retraction: MicroRNA-451 blockade promotes osteoblastic differentiation and skeletal anabolic effects by promoting YWHAZ-mediated RUNX2 protein stabilization

**DOI:** 10.1039/d1md90001c

**Published:** 2021-01-26

**Authors:** Katie Lim

**Affiliations:** a Royal Society of Chemistry , Thomas Graham House , Science Park, Milton Road , Cambridge , CB4 0WF , UK . Email: medchem-rsc@rsc.org

## Abstract

Retraction of ‘MicroRNA-451 blockade promotes osteoblastic differentiation and skeletal anabolic effects by promoting YWHAZ-mediated RUNX2 protein stabilization’ by Jieen Pan *et al.*, *Med. Chem. Commun.*, 2018, **9**, 1359–1368, DOI: ; 10.1039/C8MD00187A.



## 

The Royal Society of Chemistry hereby wholly retracts this *MedChemComm* article due to concerns with the reliability of the data. The images in the article, and the raw data provided by the authors, were screened by an image integrity expert.

The images of the cultures in Fig. 2, 3 and 5 show signs of cloning as duplicating features can be observed, which indicates that the images have been manipulated.

In addition, the raw data provided for the Western blot images does not appear to be genuine. In most of the raw images, the area immediately around the bands has a different structure or appearance to the surrounding area. Furthermore, there are traces of cloning in the background of the raw data for multiple panels including Fig. 3C (AKT), Fig. 3C (SMURF2) and Fig. 3J (p-AKT), indicating that the raw data was manipulated. Therefore, the raw data provided by the authors cannot be used to validate the published data.

Given the significance of the concerns about the validity of both the data in the article and the raw data provided by the authors, the findings presented in this paper are not reliable.

The authors have been informed but have not responded to any correspondence regarding the retraction.

 

Signed: Katie Lim, Executive Editor, *RSC Medicinal Chemistry*

Date: 15th January 2021

